# Lithium Induced Amenorrhea: A First Case Report

**DOI:** 10.15388/Amed.2024.31.1.5

**Published:** 2024-02-27

**Authors:** Balakumar KE, Vandana Tayal, Vidhya Egambarame, Vandana Roy

**Affiliations:** Maulana Azad Medical College, India

**Keywords:** female health, lithium, psychiatry, moterų sveikata, litis, psichiatrija

## Abstract

A 19-year-old female, known case of bipolar disorder had history of amenorrhea (absence of menstruation) for three years. While other causes of amenorrhea such as CNS, pregnancy, other medications, and thyroid issues were ruled out, she was found to have hyper-prolactinemia. Although antipsychotic medications are known to cause amenorrhea due to dopamine receptor blockade, which may result in hyperprolactinemia, the patient’s symptoms began before she started these medications. Only drug that she was on for long period is lithium. Current literature shows mixed evidence about lithium’s impact on prolactin levels, which can affect menstruation. This case may represent the first report of lithium causing amenorrhea through elevated prolactin levels. Clinicians should be aware of this potential side effect and monitor patients accordingly. Further studies are needed to confirm and understand this potential link.

## Abstract (it)

Devyniolikametė mergina, kuriai buvo nustatytas bipolinis sutrikimas, jau trejus metus turėjo sveikatos problemų dėl amenorėjos (nėra menstruacijų). Atmetus visas kitas amenorėjos priežastis, tokias kaip centrinės nervų sistemos problemos, nėštumas, kitų medikamentų vartojimas ar skydliaukės problemos pacientei buvo nustatyta hiperprolaktinemija. Nors yra duomenų, kad antipsichoziniai medikamentai gali sukelti amenorėją dėl dopamino receptorių blokavimo, tačiau pacientei amenorėjos simptomai prasidėjo anksčiau, nei ji pradėjo vartoti šiuos medikamentus. Vienintelis vaistas, jos vartotas ilgą laiką, buvo litis. Naujausios literatūros pateikiami duomenys apie ličio poveikį prolaktino lygiui nėra neginčytini, tačiau žinoma, kad prolaktino lygis gali turėti poveikį menstruacijoms. Tikėtina, kad šis atvejis yra pirmoji publikacija, kurioje aptariama ličio sukelta amenorėja dėl pakilusio prolaktino lygio. Gydytojams būtų naudinga žinoti apie šį galimą šalutinį poveikį, kad jie galėtų stebėti savo pacientes ir dėl šio kriterijaus. Norint patvirtinti ir visavertiškai suvokti šį ryšį, būtini tolesni tyrimai.

Secondary amenorrhea refers to the absence of three or more periods continuously in a woman who has attained puberty [1]. Pregnancy is the most common cause of secondary amenorrhea. Other causes include hyperprolactinemia due to breastfeeding, medications like antipsychotics, antidepressants, cancer chemotherapy and oral contraceptives. Pituitary tumours, hypothyroidism, polycystic ovarian syndrome (PCOS) and structural problems like Asherman’s syndrome are also associated with amenorrhea [2].

Medication induced hyperprolactinemia is the main cause of drug induced amenorrhea [3]. Antipsychotics by blockade of D2 dopamine receptors are well known to cause endocrine adverse events such as amenorrhea and galactorrhoea [4]. The prevalence of antipsychotics induced amenorrhea is reported to be 44.3% [5].

Lithium, a mood stabiliser, is used as the first line drug for the treatment of maniac episode of bipolar disorder as well as in its maintenance therapy. It is also used in clinical conditions like major depression as an adjunctive treatment, neutropenia, and bipolar disorder without mania [6].

The commonly reported adverse effects of lithium include thirst, excessive urination, nausea, diarrhoea and tremors. Other not so common but often distressing adverse effects requiring withdrawal from therapy include diabetes insipidus, hypothyroidism, hyperparathyroidism [7].

To our knowledge, no case of lithium induced amenorrhea has been reported till date.

## Case Report

Here we present a case of a 19-year-old unmarried female diagnosed with bipolar disorder since 2019. She was admitted in psychiatry ward with features of mania in the month of November 2022. Her history revealed that she had amenorrhea for the last 3 years. On admission patient was given treatment for the presenting illness and was also evaluated for amenorrhea. Patient height – 157 cm, weight – 60 kg and BMI – 24.75 kg/m2.

Her medication history revealed that the patient was on treatment with tablet lithium for bipolar disorder and was taking medication irregularly for her condition since 2019. However, the treatment records for the same were not available. As per the available records dated from November 2021, she was on tablet Sodium Valproate 200–800 mg (3 months), tablet Olanzapine 15 mg (6 months), tablet Trihexyphenidyl 2 mg (1 week). After 3 months of starting sodium valproate, patient reported to have suicidal ideation and had weight gain, she was, hence, switched back to tablet lithium 300 mg. She was also prescribed tablet Paroxetine 25 mg (3 months), tablet Aripiprazole 5 mg (1 month) during the treatment periods.

Her menstrual history revealed that the patient attained menarche at the age of 13 years and had regular menstrual cycle till 16 years of age. She complained of having amenorrhea for the last 3 years. There is no history of hypertension, diabetes mellitus, hypothyroidism or any other surgical intervention in the past.

Pregnancy test was negative and other tests to investigate organic etiology were normal. Relevant blood investigations revealed that the patient had subclinical hypothyroidism and hyperprolactinemia. The laboratory values were as follows: FT3 – 3.4 pg/ml (N – 2.0 to 4.4 pg/ml), FT4 – 1.0 ng/ml (N – 0.93 to 1.93 ng/ml), TSH – 11.2 µIU/ml (<4.2 µIU/ml), Prolactin – 2192 µIU/ml (<495 µIU/ml). Noncontrast CT head done on 22/12/2022, showed old, calcified granuloma right parietal lobe with no perilesional edema.

After ruling out possible causes of hyperprolactinemia, lithium treatment was thought to be responsible for the observed symptoms. After admission, tablet lithium was stopped, and patient was started on tablet olanzapine 10 mg. After lithium replacement serum prolactin levels were repeated and were found to be reduced to 984 µIU/ml. Two weeks after discontinuation of lithium, the patient had a menstrual period. We determined the causality using the WHO-Uppsala Monitoring Centre (UMC) causality assessment scale. The causality was “probable” according to the WHO-UMC scale and Naranjo scale (score – 8). This case was reported to the ADR monitoring centre under the Pharmacovigilance Programme of India.

## Discussion

To the best of our knowledge, this is the first case of lithium induced amenorrhea with hyperprolactinemia. The patient was evaluated first for the cause of amenorrhea. The possible reasons like pregnancy, hypothyroidism, ovarian, pituitary, and other drug related causes were ruled out. The patient had subclinical hypothyroidism, which could be possibly because of long term lithium therapy [8]. Patient also had unilocular cyst in the ovary, but these conditions are not known to cause amenorrhea [9][10]. The presence of amenorrhea was thus thought to be related to either pituitary cause or because of medication.

Pituitary cause of amenorrhea was also ruled out as the patient had no symptoms of headache, vomiting, blurring of vision etc. Additionally, noncontrast CT head findings were nonspecific.

Amenorrhea was then considered to be due to hyperprolactinemia associated with the medication use. Antipsychotics are well known to cause hyperprolactinemia and amenorrhea by blockade of dopamine receptors. However, these antipsychotics were also ruled out as a cause since amenorrhea developed much before they were prescribed to the patient.

In this patient lithium was started 3 years ago which may have resulted in the development of amenorrhea, but its mechanism of action remains poorly understood. At a neuronal level, lithium reduces excitatory (dopamine and glutamate) but increases inhibitory (GABA) neurotransmission. Since dopamine has an inhibitory effect over prolactin [11], the treatment with lithium increases prolactin levels. A study found that lithium can also increase prolactin, possibly through a decrease in dopamine receptor sensitivity [12]. Resulting hyperprolactinemia can lead to amenorrhea, and prolonged amenorrhea in association with hyperprolactinemia incurs significant risks for bone health in adolescent girls.

However, the effects of lithium on prolactin secretion are controversial and wide inter-individual variations have been observed [11][12].

A longitudinal follow-up study done with 9 depressive patients and a transversal study in 26 patients showed lithium has no effect on prolactin levels as compared to control group [13]. Another study revealed that the role of lithium in prolactin release is correlated with the duration of therapy. It stated that treatment with lithium for less than six months does not result in any change in the secretion of prolactin, whereas a treatment of more than 6 month decreases the level of prolactin as compared to control group [14].

In an earlier case report, the development of hyperprolactinemia as a consequence of treatment with lithium in combination with aripiprazole (15 mg/day) in a 18-year-old girl with bipolar disorder has been reported [15]. However, in that patient the subsequent replacement of aripiprazole with quetiapine resulted in alleviation of hyperprolactinemia.

In our patient, lithium was stopped upon admission and a subsequent reduction in serum prolactin after 1 week was observed and the patient resumed menstruation after 2 weeks. These events indicate that the amenorrhea was due to lithium. The patient was not rechallenged with lithium.

Our objective of reporting this case is to highlight the concept of lithium causing hyperprolactinemia and thus amenorrhea. This can be taken as the first reported case of the same. Disruption of normal endocrine function can have serious long-term consequences. Therefore, clinicians need to be familiar with this rare but possible adverse effect of lithium and be prepared to implement appropriate monitoring and management strategies.
